# Evaluating the impact of biannual school-based and community-wide treatment on urogenital schistosomiasis in Niger

**DOI:** 10.1186/s13071-020-04411-9

**Published:** 2020-11-18

**Authors:** Anna E. Phillips, Zilahatou Tohon, Neerav A. Dhanani, Boubacar Sofo, Issa Gnandou, Boubacar Sidikou, Adamou Garba Noma, Bassirou Madougou, Oumarou Alto, Hannatou Sebangou, Kader M. Halilou, Roumanatou Andia, Amadou Garba, Alan Fenwick, Amina A. Hamidou

**Affiliations:** 1 London Centre for Neglected Tropical Disease Research (LCNTDR), Department of Infectious Disease Epidemiology, Imperial College London, London, UK; 2 Aménagement et Lutte (RISEAL NIGER), Réseau International Schistosomiases Environnement, Avenue de l’indépendance, BP. 13724, Niamey, Niger; 3 Schistosomiasis control Initiative (SCI), Department of Infectious Disease Epidemiology, Imperial College London, London, UK; 4 Ministère de la Santé, Niamey, Niger; 5 Hôpital National de Niamey rond-point Hôpital, BP 238, Niamey, Niger

**Keywords:** Schistosomiasis, Urogenital schistosomiasis, Community-wide treatment, School-based treatment, Biannual treatment, Elimination

## Abstract

**Background:**

The Schistosomiasis Consortium for Operational Research and Evaluation (SCORE) coordinated a five-year study implemented in several countries, including Niger, to provide an evidence-base for programmatic decisions regarding cost-effective approaches to preventive chemotherapy for schistosomiasis control.

**Methods:**

This was a cluster-randomised trial investigating six possible combinations of annual or biannual community-wide treatment (CWT), school-based treatment (SBT), and holidays from mass treatment over four years. The most intense arm involved two years of annual CWT followed by 2 years of biannual CWT, while the least intensive arm involved one year of annual SBT followed by a year without treatment and two more years of annual SBT. The primary outcome of interest was prevalence and intensity of *Schistosoma haematobium* among 100 children aged 9–12 years sampled each year. In addition, 100 children aged 5–8 years in their first year of school and 50 adults (aged 20–55 years) were tested in the first and final fifth year of the study.

**Results:**

In total, data were collected from 167,500 individuals across 225 villages in nine districts within the Niger River valley, Western Niger. Overall, the prevalence of *S. haematobium* decreased from baseline to Year 5 across all study arms. The relative reduction of prevalence was greater in biannual compared with annual treatment across all arms; however, the only significant difference was seen in areas with a high starting prevalence. Although adults were not targeted for treatment in SBT arms, a statistically significant decrease in prevalence among adults was seen in moderate prevalence areas receiving biannual (10.7% to 4.8%) SBT (*P* < 0.001). Adults tested in the annual SBT group also showed a decrease in prevalence between Year 1 and Year 5 (12.2% to 11.0%), but this difference was not significant.

**Conclusions:**

These findings are an important consideration for schistosomiasis control programmes that are considering elimination and support the idea that scaling up the frequency of treatment rounds, particularly in areas of low prevalence, will not eliminate schistosomiasis. Interestingly, the finding that prevalence decreased among adults in SBT arms suggests that transmission in the community can be reduced, even where only school children are being treated, which could have logistical and cost-saving implications for the national control programmes.

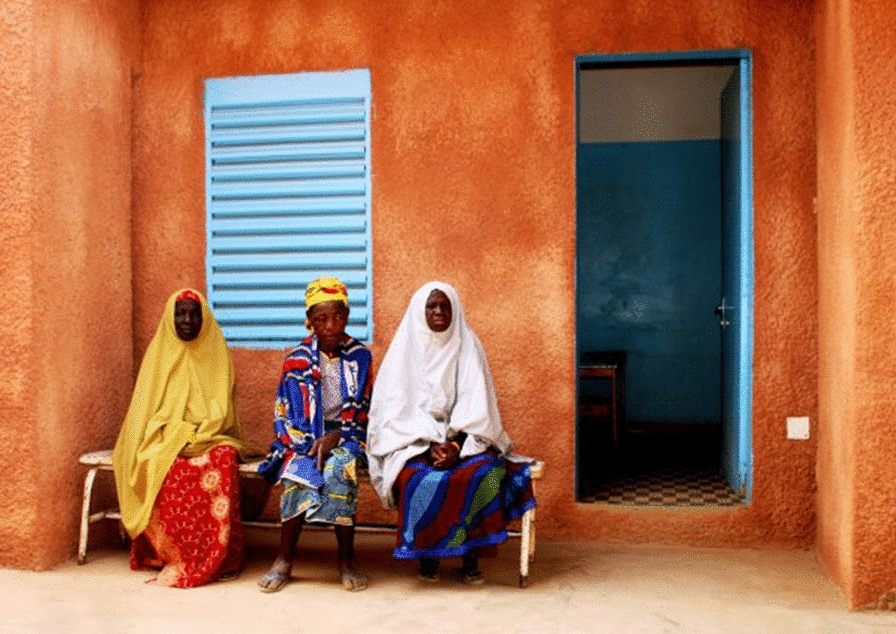

## Background

Human schistosomiasis is an acute and chronic, water-associated parasitic disease that remains a major public health problem in sub-Saharan Africa. It represents the second most endemic parasite after malaria in these regions [1]. According to the World Health Organisation (WHO), an estimated 218 million people need preventive chemotherapy (PC) worldwide, of which 92% live in Africa [2].

It is estimated that 3.2 million people are infected with schistosomiasis in Niger [3]. Both *Schistosoma haematobium* (urogenital) and *Schistosoma mansoni* (intestinal) are endemic, but the main species is *S. haematobium*, which is distributed in all regions of the country [4]. Previously *S. mansoni* had a relatively marginal role; however, more recently an increase in infection has been seen in the western part of the Niger River Valley. A national programme for the control of schistosomiasis and geohelminthiasis was implemented by the Niger Ministry of Health in 2004 [5]. The control strategies used were school-based treatment (SBT) with praziquantel (PZQ) and selective chemotherapy in adults at high risk of infection, following the WHO guidelines [6].

Previously, a combined school- and community-based strategy has been shown to be effective in attaining a high coverage among school-aged children (SAC) in countries where school enrolment is low and where schools cannot serve as the exclusive drug distribution points [7–10]. There is a growing body of evidence regarding the burden of infection in adults and their potential role in sustaining transmission, which suggests a need for them to be included in treatment programmes [10–15].

Whether community-wide treatment (CWT) is appropriate depends on the local epidemiological setting and whether the goal is morbidity control or eliminating transmission [16]. Some studies, for example, have found that SBT and community-wide treatment (CWT) yielded a similar prevalence decrease [17, 18]. There is also debate around the optimal frequency of PZQ treatment for infection and morbidity control where some studies have found that the more frequent the PC, the greater impact on parasite control [19], while others have shown that a single dose of PZQ results in sustained low transmission of *S. haematobium* for 2 years [20]. Considering the controversy around the optimal approach to drug-based control of schistosomiasis, the Schistosomiasis Consortium for Operational Research and Evaluation (SCORE) was established in 2008 to address this and other questions of practical significance. An important part of SCORE’s portfolio was multi-country field studies, which included Niger, that have evaluated the impact of alternative approaches to PC through CWT, SBT, and years without mass drug administration (MDA) [21].

The SCORE protocol and baseline characteristics of study populations have been described elsewhere [22, 23]. In brief, SCORE projects include “Gaining control of schistosomiasis” studies, evaluating PC in communities with high prevalence of schistosomiasis, and “Sustaining control of schistosomiasis” studies, examining PC in areas of moderate schistosomiasis prevalence [21].

In 2011, SCORE began supporting Niger to conduct both “Gaining” and “Sustaining” *S. haematobium* control studies in two separate parts of the Niger River Valley. A simple random allocation approach was supposed to have been used to assign all eligible villages to study arms. Instead, during the selection of the field sites, Niger geographically clustered groups of villages into study arms. Following Year 2 of the study, when this was recognised, the Niger study was redesigned to compare annual versus biannual SBT or CWT. An additional deviation from the SCORE protocol included replacing the treatment ‘holiday’ years with a test-and-refer strategy, whereby mass drug administration was not given on these years, but individuals were tested and those found to be positive were referred to health centres for treatment.

The primary research question presented here is which PC strategy provided the greatest reduction in prevalence and intensity of *S. haematobium* infection among 9–12 year-olds after four years of intervention. In addition, the impact of treatment on first-year students and adults was also assessed.

## Methods

### Study design

SCORE implemented a parallel cluster-randomised intervention trial that includes six study arms for the “Gaining” control study and three arms for the “Sustaining” control study [21]. The primary outcome of interest was the prevalence and intensity among 9–12 year-old children following four years of MDA using different approaches. In Niger, the “Gaining” study was to be implemented in an area that historically had high levels of infection, and the “Sustaining” study in a nearby area expected to have lower transmission.

**Fig. 1:**
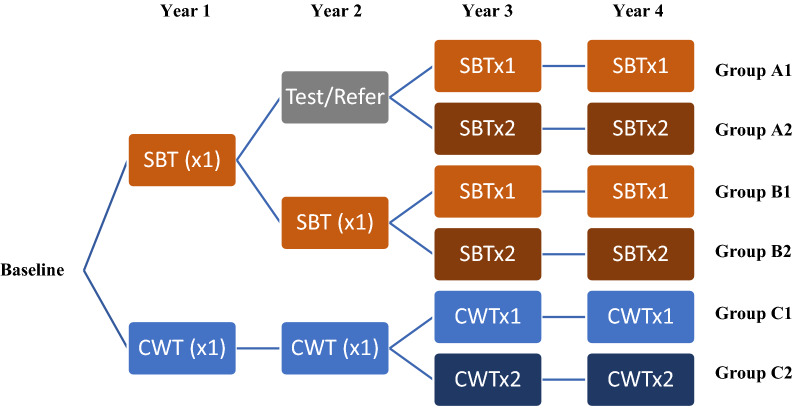
Niger SCORE study design combining “Gaining” and “Sustaining” studies. There are six study arms, where by each arm received a four-year treatment strategy with varying combinations of annual school-based treatment (SBTx1), biannual school-based treatment (SBTx2), annual community-wide treatment (CWTx1), biannual community-wide treatment (CWTx2), and “Test & Treat” (i.e. where test and referral for treatment): Arm 1: SBTx1-Test & Treat-SBTx1-SBTx1; Arm 2: SBTx1-Test & Treat-SBTx2-SBTx2; Arm 3: Annual SBT; Arm 4: SBTx1-SBTx1- SBTx2- SBTx2; Arm 5: Annual CWT; Arm 6: Biannual CWT

The original design relied on randomisation so that starting prevalence would be roughly equivalent in different study arms. However, because of the way in which the Niger study clustered villages, the starting prevalence were markedly different in different arms, so valid comparisons between arms and comparison with other SCORE studies could not be made. Therefore, in 2013 (before Year 3 of the study) the Niger study was re-designed to evaluate the impact of either SBT or CWT twice a year (biannual) versus annual treatment with PZQ. Thus, the communities received a variable sequence of SBT, CWT or test/refer for treatment strategies for the first two years, and then once or twice-yearly treatment for the final two treatment years, with the final round of data collection done 12 months after the final (Year 4) treatment round. The reformatted study design, upon which the following analyses are based, is summarised in Fig. 1.

The 75 communities found eligible with 10–24% prevalence initially received either SBT in Years 1 and 2, or SBT in Year 1 and were tested and referred for treatment in Year 2. Before Year 3 these were combined and re-randomised into Arm A1, which received SBT once a year in Years 3 and 4, or Arm A2, which received SBT twice a year in Years 3 and 4. Of the communities found eligible with ≥ 25%, 75 received either SBT in Years 1 and 2, or SBT in Year 1 and were tested and referred for treatment in Year 2. Before Year 3 these were combined and re-randomised into Arm B1, which received SBT once a year in Years 3 and 4, or Arm B2, which received SBT twice a year in Years 3 and 4. The other 75 communities found eligible with ≥ 25% received CWT in Years 1 and 2 and before Year 3 were re-randomised into Arm C1, which received CWT once in Years 3 and 4 and Arm C2 which received CWT twice a year in Years 3 and 4 (Fig. 1).

The number of villages per arm and number of 9–12 year-old students tested per village in the original study design were based on sample size calculations [21]. Based on these calculations, a village’s prevalence and intensity of *S. haematobium* infections were monitored each year among a random sample of 100 school children aged 9–12 years. A total of 225 villages were enrolled, with 22,500 children aged 9–12 years tested each year. In addition, systematic random sampling was used in each village to select a further 100 first-year students (aged 5–8 years) in all 225 villages at the first and fifth years only. A convenience sample of 50 adults (aged 22–50 years) were sampled in the first and last year of the “Gaining” study.

Communities were only eligible to participate in the study if they had a primary school with at least 100 school children aged 9–12 years and if they met the prevalence criteria for either “Gaining” or “Sustaining” control studies. Prevalence was assessed by testing fifty children aged 13–14 years in each potential study community using reagent Hemastix® testing for microhaematuria on a single midday urine sample [24]. Trace results were considered positive per manufacturer instructions. Communities in the area selected for the “Gaining” studies were eligible if they had high prevalence (≥ 25%), and for “Sustaining” studies if they had moderate prevalence (10–24%) among 13–14 year-olds in the eligibility assessment (the 13–14 year-old age group was used to assess eligibility because of the need to treat children testing positive, which would have affected baseline study results).

The 150 communities found eligible for “Gaining” were initially assigned to one of six arms of the original study design and the 75 villages eligible for “Sustaining” were allocated to one of three study arms in the original study design. When the study was re-designed, each arm’s villages were randomly assigned to once- *vs* twice-a-year treatment using a computer-based randomisation. Thus, communities received a variable sequence of CWT, SBT, or test/refer for treatment strategies for the first two years, and then once or twice-yearly treatment for the final 2 years, with the final round of data collection carried out 12 months after the final (Year 4) treatment round in Year 4.

### Study area and population

**Fig. 2:**
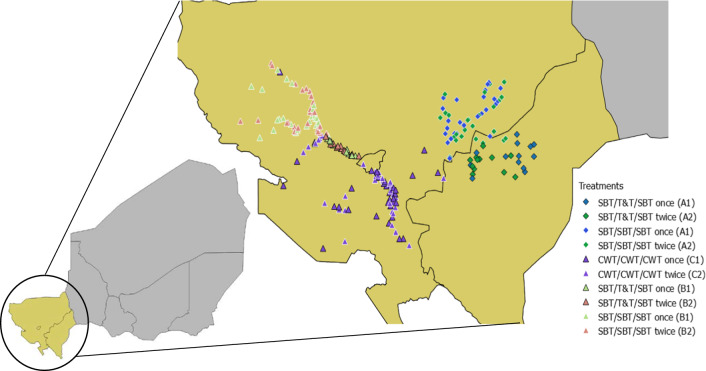
Map of the study zone. The site of each village has been colour-coded on the map according to the study arm

The study was conducted in the region of Tillabery (Kollo, Say, Tera, Filingué and Tillabery districts), Dosso (Loga district) and Niamey (districts 1-3) in Western Niger along the Niger River Valley (Fig. 2). The climate is characterised by a short rainy season from June to September and a long dry season from October to May. Eight districts are only endemic to *S. haematobium*, but the district of Tillabery is endemic to *S. haematobium* and to a lesser extent *S. mansoni*. The main sites for transmission are irrigation canals, rice paddies and temporary water bodies that fill up during the rainy season. The main occupations of the population are agriculture and livestock. Millet is grown during the rainy season, while rice is grown in the Niger River valley twice a year. PC has been implemented in the study area since 2004 by the national schistosomiasis control programme. Mass treatment with praziquantel has been provided more than eight times with acceptable coverage rates [5]. Although schooling is compulsory in principle for ages 7–15 years-old for a period of eight years, there is only about a 25% school attendance by primary school-age children, and even fewer 12–17 year-olds continue to secondary school [25]. Sanitation in the study area is also low; over 20% of deaths in Niger can be attributed to poor sanitation and hygiene [26]. While Niger has one of the lowest sanitation coverage rates in the world, access to improved sanitation has increased over the last ten years. However, Niger has one of the highest population growth rates in the world, which has likely blunted the impact of some of the progress being made [27, 28].

### Study participants

The 9–12 year-old children were selected based on a list of students provided by the teacher with the name, sex and age of each child in the class, with younger children being selected first and recruitment continuing until the target number was achieved. In schools where less than 100 children were available, community leaders encouraged parents of non-school attending children to volunteer and enrol in the survey. All children were given a container in which to provide a single urine sample collected between 10:00 and 14:00 h. A similar approach was used to select first-year students who were 5–8 years-old for testing in Years 1 and 5. Convenience samples of adults were recruited to participate in the study by presenting at the school on the day of data collection on a voluntary basis as a convenience sample.

### Urine examination

A single urine was collected from each individual in the study for assessment of parasitological outcomes, two filtrations were carried out on two 10 ml aliquots of each urine sample using a Nytrel® mesh according to the method of Plouvier et al. [29]. One filtration was examined immediately in the school, and the second filtration by another technician in the national schistosomiasis laboratory. Both filters were coloured with lugol and *S. haematobium* eggs were counted under a light microscope. Intensity of infection was defined as negative (no eggs found), light (1–49 eggs/10 ml), and heavy (≥ 50 eggs/10 ml) infection [6]. Number of eggs counted was capped at 1000 eggs/10 ml. Where urine volume was less than 10 ml, the volume of urine was measured, and the number of eggs recalculated per 10 ml. The mean number of eggs of child was calculated as the arithmetic mean of the two filtrations taken from a single urine sample, including both egg positive and negative. A person was deemed egg-positive if one or more eggs were found in any of the slides examined.

### Treatment

Prior to treatment, community sensitisation was carried out using “public criers” and local radios to inform and mobilise the population to participate. In SBT villages, children were treated with a single dose of PZQ regardless of parasitological results [21], unless they met the WHO treatment exclusion criteria [6]. In villages receiving CWT, the entire eligible population were treated, with only children under 5 years of age or under 94 cm in height excluded [6]. In test/refer for treatment villages, children who tested positive were referred to health centres for treatment. The dose of PZQ for children was determined using a WHO dose pole [30].

In CWT, drugs were distributed door-to-door by trained community drug distributors (CDDs). All CDDs were selected by the community and trained by the local health facility nurse prior to each treatment campaign. In SBT, PZQ was administered by trained teachers to all primary school-aged children, both enrolled and non-enrolled, regardless of infection status. Training and supervision for both CWT and SBT were provided each year prior to the MDA by local health workers. The importance of directly observed therapy was emphasised at training, which was closely monitored particularly in schools as the survey teams collected urine samples on the same day (prior) to treatment. In the communities, the CDD were spot checked to ensure that compliance was adhered to. Coverage was estimated using the national population census estimates as a denominator. Where coverage was less than 75%, additional “mop-up” efforts were made to increase the coverage. Treatment numbers were recorded on registers and collected at the end of the MDA by the health workers and transmitted to the national control programme. Any secondary adverse effects were reported.

### Data management and analysis

All demographic and laboratory data were entered on paper forms and double-entered into Microsoft Excel. Data cleaning and management was carried out by RISEAL Niger. Data analysis was performed using R version 3.2 [31]. The parasitological results were averaged across each village to produce a village level prevalence and village level prevalence of heavy infected, respectively. 95% confidence intervals were calculated for village- and study arm-level prevalence values. Prevalence of infection and heavy infection were calculated as arithmetic means of the infection categories, aggregated by the relevant factors (e.g. age, sex). The arithmetic mean of infection intensity was calculated using both egg-positive and negative, aggregated by grouping factors (village, study arm, and sex). Although mean intensity might have been underestimated as egg counts were capped at 1000 eggs/10 ml, this was done consistently over the years. Therefore, the trend in intensity change is believed to be valid. The egg reduction rate was calculated as the reduction in the intensity of infection assessed indirectly, using egg count *via* the following formula: % egg reduction = 100 × (1 − (Arithmetic mean of eggs/10 ml urine after treatment in Year 5 / Arithmetic mean of eggs/10 ml urine at baseline)).

Village-level prevalence was calculated as the percent of children having at least one egg detected by urine filtration. Once and twice-yearly treatment were compared using a generalised linear mixed model (GLMM). The models were run on three subsets of the data, the 9–12 year-old cross-section, 20–55 year-olds (adult) and 5–8 year-olds. The *lme4* package was used in R and the function glmer to model the difference in the chance of being infected at the end of the study between treatment groups. The parameter being modelled was a binary factor to indicate infection status (0 = not infected, 1 = infected). Treatment group, sex and age were included as fixed effects and village was included as a random effect. To avoid issues of multiple testing we focused on group comparisons. In Years 3 and 4: Group A in low prevalence areas received SBT once a year (A1), and twice a year (A2); Group B in high prevalence areas receiving SBT once a year (B1), and twice a year (B2); and Group C in high prevalence areas receiving CWT once a year (C1), and twice a year (C2). For Group C, the prevalence at the start of the study was significantly different between villages in C1 and those in C2. Therefore, baseline data were included in the analysis and the model was adjusted to include study year and an interaction term between study year and treatment group as fixed effects.

## Results

### Eligibility survey

The eligibility survey was conducted between October 2010 and January 2011. In total 19,990 children aged 13–14 years were randomly selected from 348 villages (where 50 children were randomly selected and tested in each school) and screened for haematuria using urine dipsticks. Overall, 150 villages were screened in moderate endemicity areas for the “Sustaining” control study, of which 75 met the criteria (haematuria prevalence of 10–24%). A total of 248 villages were screened for the high prevalence “Gaining” control study, of which 150 met the criteria (haematuria prevalence ≥ 25%).

### Treatment coverage

Drug coverage was defined as the proportion of individuals who were directly observed to have ingested the PZQ. CWT coverage was determined by the total population treated using estimated projections of the denominator of the whole population based on a population census carried out in 2011, which is also used by the National Schistosomiasis and STH control programme to calculate treatment coverage. The SBT coverage was determined by the percentage of all school-aged children (5–12 years-old) that were treated as a proportion of school-aged children estimated in the population census, and therefore included children not enrolled in school. Treatment coverage is summarised in Table 1.

**Table 1:** Treatment coverage by Group over time

Year (Y)	Study group	SAC treated	SAC total	Mean % SAC treated^a^	Total population treated	Total population eligible for treatment	% total population treated
Y1	A1	16,563	20,267	82			
	A2	18,986	18,809	101			
	B1	20,382	24,670	83			
	B2	25,281	30,373	83			
	C1	22,479	17,598	128	43,600	58,648	74
	C2	26,373	25,133	105	57,058	83,772	68
Y2	A1	15,053	13,425	112			
	A2	13,553	14,309	95			
	B1	19,322	17,378	111			
	B2	16,345	17,227	95			
	C1	23,512	18,123	130	47,031	60,410	78
	C2	33,735	25,883	130	66,671	86,287	77
Y3 Treatment 1	A1	23,281	23,021	101			
	A2	25,174	23,586	107			
	B1	28,406	22,999	124			
	B2	33,231	27,781	120			
	C1	26,306	19,663	134	51,598	52,434	98
	C2	39,311	24,415	161	74,558	65,106	115
Y3 Treatment 2	A1	0	23,021				
	A2	28,203	23,586	120			
	B1	0	22,999				
	B2	25,274	27,781	91			
	C1	0	19,663			52,434	
	C2	33,003	24,415	135	61,182	65,106	94
Y4 Treatment 1	A1	20,708	25,368	82			
	A2	18,178	21,528	85			
	B1	27,316	29,813	92			
	B2	29,202	33,822	86			
	C1	22,664	19,042	120	42,464	50,761	84
	C2	33,861	25,866	131	55,831	68,967	81
Y4 Treatment 2	A1	0	25,368				
	A2	16,309	21,528	76			
	B1	0	29,813				
	B2	26,247	33,822	78			
	C1	0	19,042			50,761	
	C2	29,290	25,866	113	54,539	68,967	79

^a^Arithmetic mean weighted by village SAC population

The nominal treatment coverage stipulated by the study protocol was coverage of more than 75% for both SBT and CWT. Overall, there was extremely high coverage across all arms, this was expected the communities enrolled in this community are rural and isolated. However, in some Groups there is reported coverage above 100%. This could be explained by an inaccurate denominator, migration into the community for treatment, and treatment of adults receiving treatment during the SBT. When coverage was included into the multivariate model looking at impact on changes of prevalence over time, there was no clear trend or significant relationship with impact on infection.

### Summary sample characteristics by study year for all individuals examined

Data were collected in the dry season between October and February each year from 2011 until 2015 across nine districts in the Niger River Valley. The aim was to sample 167,500 individuals from a total of 225 villages over the five years. In total 166,811 individuals were tested.

Table 2 shows the sample characteristics by study year, by age group. Overall, nearly half of individuals sampled were female consistently across all study years and age groups.

**Table 2:** Summary of sample characteristics by study year for all individuals examined (5–8 years-old, 9–12 years-old, and adults)

Cross-section	Variable	Baseline (2011)	Year 2 (2012)	Year 3 (2013)	Year 4 (2014)	Year 5 (2015)
5−8 years	No. of individuals sampled	20,220				22,364
	Proportion female (%)	9328 (46.1)				10,227 (45.8)
	No. infected with *S. haematobium* (%)	3474 (17.2)				2134 (9.54)
	No. with heavy intensity of infection (%)	391 (1.93)				239 (1.07)
	Arithmetic mean egg count^a^	1.08				0.62
9–12 years	No. of individuals sampled	20,931	21,833	21,620	21,715	22,132
	Proportion female (%)	9438 (45.1)	9697 (44.4)	9771 (45.2)	9943 (45.8)	10,281 46.5)
	No. infected with *S. haematobium* (%)	3314 (15.8)	2089 (9.57)	3809 (17.6)	1794 (8.26)	2190 (9.89)
	No. with heavy intensity of infection (%)	276 (1.32)	99 (0.45)	292 (1.35)	146 (0.67)	146 (0.66)
	Arithmetic mean egg count^a^	3.05	1.27	3.27	1.42	1.45
Adults (20–55 years)	No. of individuals sampled	7041				9,955
	Proportion female (%)	3966 (56.3)				4789 (48.1)
	No. infected with *S. haematobium* (%)	793 (11.3)				493 (4.95)
	No. with heavy intensity of infection (%)	35 (0.50)				28 (0.28)
	Arithmetic mean egg count^a^	4.61				2.05

^a^The mean egg count among all tested subjects (including those with zero egg counts), which is a measure of community level contamination potential

### 9–12 year-olds cross-section

Over five years, 108,231 independent observations were taken from children aged 9–12 years and the prevalence and intensity of the infections in this group were designated as the primary outcome of the study. Table 3 shows the change in *S. haematobium* infection between Baseline and Year 5 in low prevalence areas treated once and twice a year with SBT only (Group A), SBT in high prevalence areas (Group B), and CWT in high prevalence areas (Group C). As expected, the relative reduction of prevalence is greater in biannual treatment compared with annual MDA across all study arms. The biggest difference is seen in arms starting in high prevalence areas, C2 and B2 receiving biannual treatment where absolute difference in prevalence between Year 5 and baseline was 12.9% and 9.1%, respectively. The egg reduction rate follows the same pattern as the prevalence rate, again with a decrease in intensity of infection at both the village-and individual-level across all study arms. Group B shows the largest difference in the egg reduction rate between the treatment strategies (72.0% at baseline to 12.0% in Year 5).

**Table 3:** Summary of *S. haematobium* infection by study arm from baseline to Year 5 (9–12 years-old cross-section only)

Variable	Group^a^						Total
	A1	A2	B1	B2	C1	C2	
No. of villages sampled	38	37	37	38	37	38	225
No. tested at baseline	3461	3221	3659	3671	3357	3562	20,931
No. infected at baseline	129	148	898	825	480	834	3314
Prevalence at baseline (%)	3.7	4.6	24.5	22.5	14.3	23.4	15.8
Prevalence of heavy infection at baseline (%)	0.29	0.28	1.86	1.93	0.86	2.50	1.29
No. tested at Year 5	3710	3648	3621	3740	3689	3724	22,132
No. infected at Year 5	22	9	839	503	425	392	2190
Prevalence at Year 5 (%)	0.6	0.2	23.2	13.4	11.5	10.5	9.90
Prevalence of heavy infection at Year 5 (%)	0.13	0.03	1.77	0.64	0.57	0.83	0.66
Absolute difference in prevalence at Year 5 and baseline	−3.1	−4.4	−1.3	−9.1	−2.8	−12.9	−5.90
Relative difference in prevalence at Year 5 and baseline (% change)	−83.8	−95.7	−5.3	−40.4	−19.6	−55.1	−37.3
Village-level arithmetic mean infection intensity at baseline^b^	0.77	0.52	4.11	5.1	1.78	5.37	2.94
Village-level arithmetic mean infection intensity at Year 5^b^	0.14	0.05	3.62	1.39	1.37	2.18	1.46
Egg reduction rate (1-Year 5 intensity/baseline)	0.82	0.9	0.12	0.73	0.23	0.59	0.57
Individual-level arithmetic mean infection intensity at baseline^c^	0.84	0.58	4.05	4.91	1.85	5.64	2.98
Individual-level arithmetic mean infection intensity at Year 5^c^	0.14	0.05	3.61	1.38	1.37	2.2	1.46

^a^Study arms: Low prevalence areas receiving SBT once a year (A1), and twice a year (A2); high prevalence areas receiving SBT once a year (B1), and twice a year (B2); and high prevalence areas receiving CWT once a year (C1), and twice a year (C2)

^b^Village-level intensity: This is the mean egg count for all tested 9–12 year-old subjects (including those with zero egg counts), which is a measure of community level contamination potential

^c^Individual-level intensity: This is the mean egg count among egg-positive 9–12 year-old subjects, which is an estimate of the intensity of infection among known active cases

**Fig. 3:**
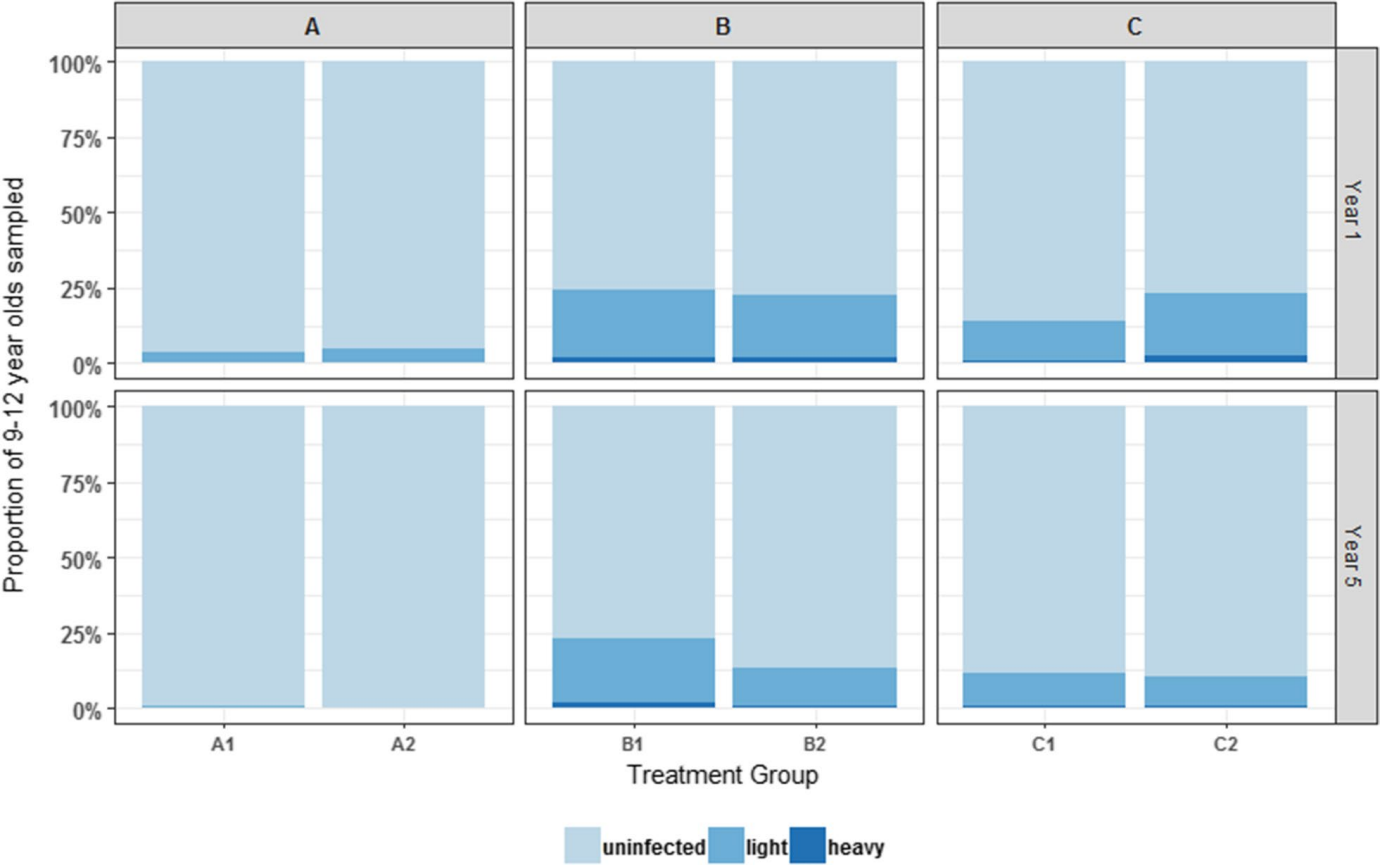
*Schistosoma haematobium* infection status by treatment group from baseline to Year 5 (9–12 year-olds cross-section only). The change in overall prevalence of *S. haematobium* infection over time from the start of the project at baseline to the final Year 5 by treatment group among the 9–12 year-old age group only is shown

Figure 3 shows the change in overall prevalence of *S. haematobium* infection over time from the start of the project at baseline to the final Year 5 by treatment group. The starting prevalence of infected individuals in Group A is low at baseline in both A1 and A2 (3.7% and 4.6%, respectively) falling to nearly 0% in Year 5. The GEE shows no statistical difference between the arms at Year 5 for Group A. Group B had a higher prevalence at baseline in both B1 and B2 (24.5% and 22.5%, respectively), with a reduction in Year 5 (23.2% and 13.4%, respectively). The GEE showed that the difference in prevalence within Group B at Year 5 was significant with B2 being lower than B1 (*P* < 0.037). Group C had a prevalence of 14.3% in C1 and 23.4% in C2 with prevalence in Year 5 being 11.5% and 10.5%, respectively. There was no statistically significant difference between C1 and C2 at Year 5.

**Fig. 4:**
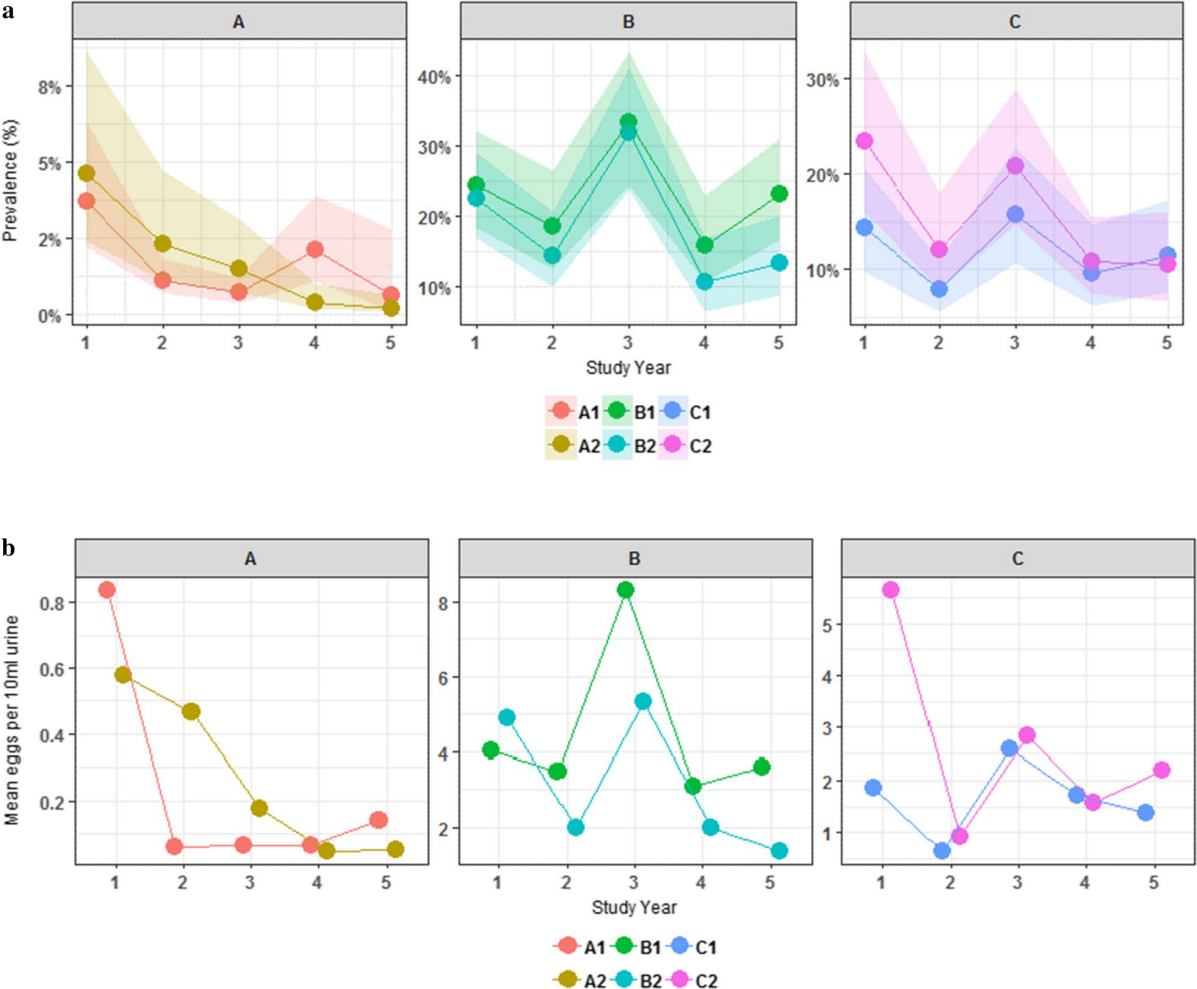
Prevalence **a** and mean intensity **b** of *Schistosoma haematobium* infection status by study arm over time (9–12 year-olds cross-section only). The change in overall prevalence and mean intensity of *S. haematobium* at each time point from the start of the project at baseline to the final Year 5 of the study is shown

Figure 4 shows the change in overall prevalence and mean intensity of *S. haematobium* at each time point from the start of the project at baseline to the final Year 5 of the study. In all arms, there was a decrease in prevalence and mean intensity over time. In Groups B and C, however, there was a peak in prevalence and intensity of infection in Year 3.

**Fig. 5:**
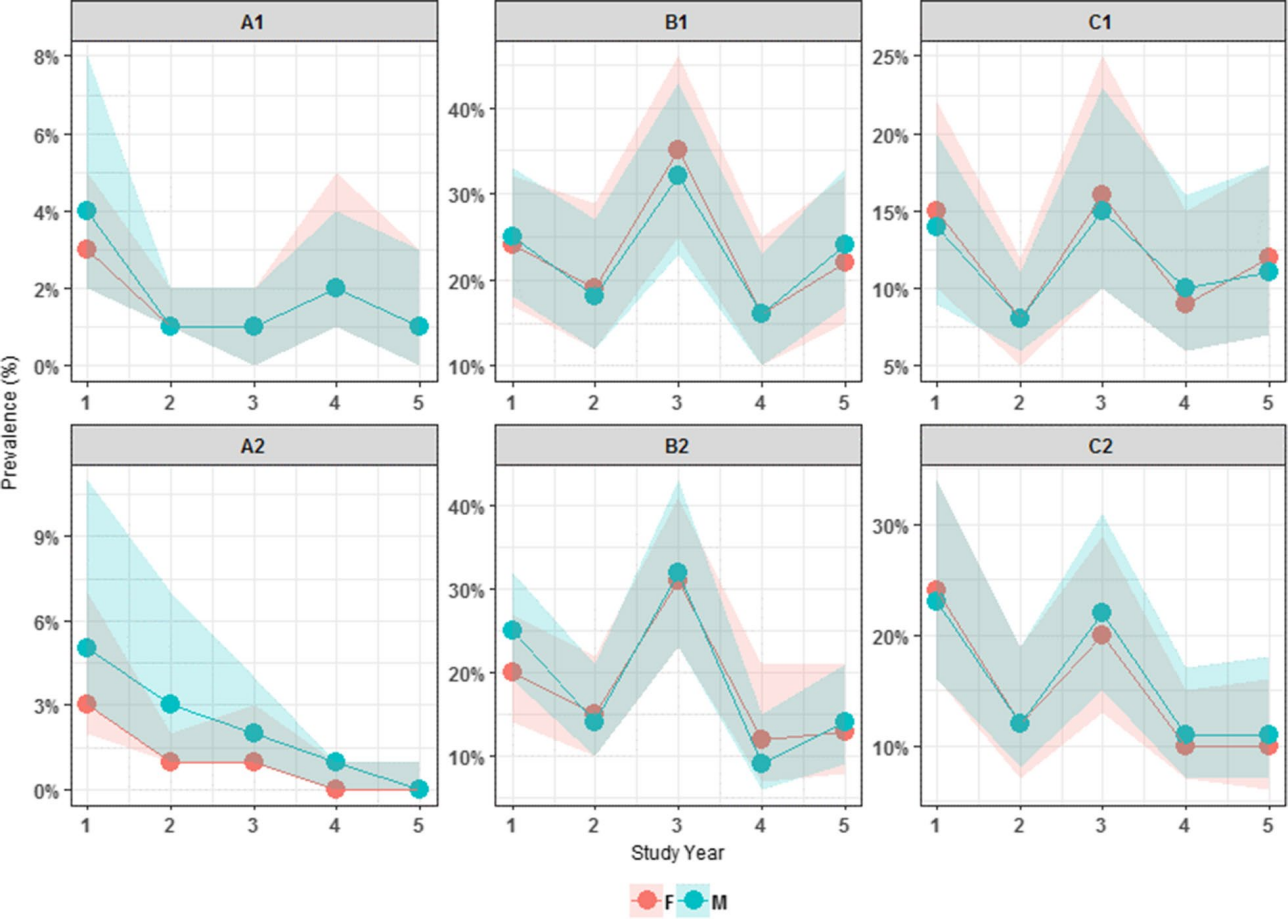
Prevalence of *Schistosoma haematobium* infection by sex (blue, boys; red, girls) over time (9–12 year-olds cross-section only)

Prevalence by sex showed a higher prevalence of infection among boys than girls, which was significant in Group B only (*P* < 0.001) (Table 4). Response to treatment was similar by sex, with both males and females showing a reduction of prevalence over time from baseline to Year 5 in most groups (Fig. 5). There was, however, a peak in prevalence of *S. haematobium* infection at Year 3 in both boys and girls in Groups B and C.

**Table 4:** Adjusted odds ratios from GLMM multivariate logistic regression model of *S. haematobium* infection at Year 5 only (*n* = 108,231 observations)

Study group	Variable	Category	Parameter	Adjusted ORs^a^	95% CI	*P*-values
Group A	Age	10 years	−1.09	0.34	0.11–1.01	*0.052*
		11 years	−1.00	0.37	0.11–1.20	0.098
		12 years	−0.81	0.44	0.17–1.18	0.104
	Sex	Male	0.07	1.07	0.48–2.35	0.871
	Treatment	Biannual treatment	0.58	1.79	0.1–33.42	0.698
Group B	Age	10 years	−0.17	0.84	0.69–1.03	0.090
		11 years	−0.28	0.75	0.61–0.94	*0.011*
		12 years	−0.48	0.62	0.5–0.77	*< 0.001*
	Sex	Male	0.26	1.30	1.13–1.50	*< 0.001*
	Treatment	Biannual treatment	−0.84	0.43	0.2–0.95	*0.037*
Group C	Age	10 years	0.03	1.03	0.83–1.28	0.815
		11 years	−0.17	0.84	0.65–1.08	0.178
		12 years	−0.30	0.74	0.57–0.95	*0.019*
	Sex	Male	0.09	1.10	0.93–1.29	0.272
	Treatment	Biannual treatment	0	1	0.43–2.33	0.999

^a^95% CIs are based on empirical standard errors

Reference groups: Age (9 years); Sex (female); Treatment (annual treatment). Statistically significant *P*-values are indicated in italics

The adjusted ORs of *S. haematobium* infection from the GLMM multivariate logistic regression models are presented in Table 4. Significance testing to assess whether the prevalence of *S. haematobium* varied over time between Groups showed that sex and age had a significant effect on infection status (Table 3). The model shows that individuals who are male are almost 1.5 times more likely than females to be infected in Group B (AOR: 1.3, *P* **<** 0.001). Children of 9 years old were more likely to be infected than older children in the 9–12 year-olds cross-section. This was significant in Group A with 10 year-olds (AOR: 0.34, *P* = 0.052); in Group B with 11 (AOR: 0.75, *P* **=** 0.011) and 12 year-olds (AOR: 0.62, *P* **<** 0.001) and Group C with 12 year-olds only (AOR: 0.74, *P* **=** 0.019). The impact of biannual versus annual treatment was also assessed with the GLMM model, where twice yearly treatment had a significantly greater impact in reducing prevalence of infection in Group B only (AOR: 0.43, *P* = 0.037).

### First-year students and adult cross-sections

In Year 1 and 5 of the study, an additional 42,584 and 16,996 observations were recorded for first-year students (aged 5–8 years) and adults (aged 20–55 years), respectively (Table 1). Overall, prevalence and intensity of infection decreased in both cross-sections over time. At baseline, examination revealed that prevalence was higher among first-year students (17.2%) compared to 11.3% of adults infected with *S. haematobium*, which fell to 9.54% and 4.95% in Year 5, respectively. The proportion of heavily infected individuals was low even at baseline, with 1.93% first-year students and 0.5% of adults, decreasing to 1.07% and 0.28% heavy infection in Year 5, respectively (Table 1).

**Fig. 6:**
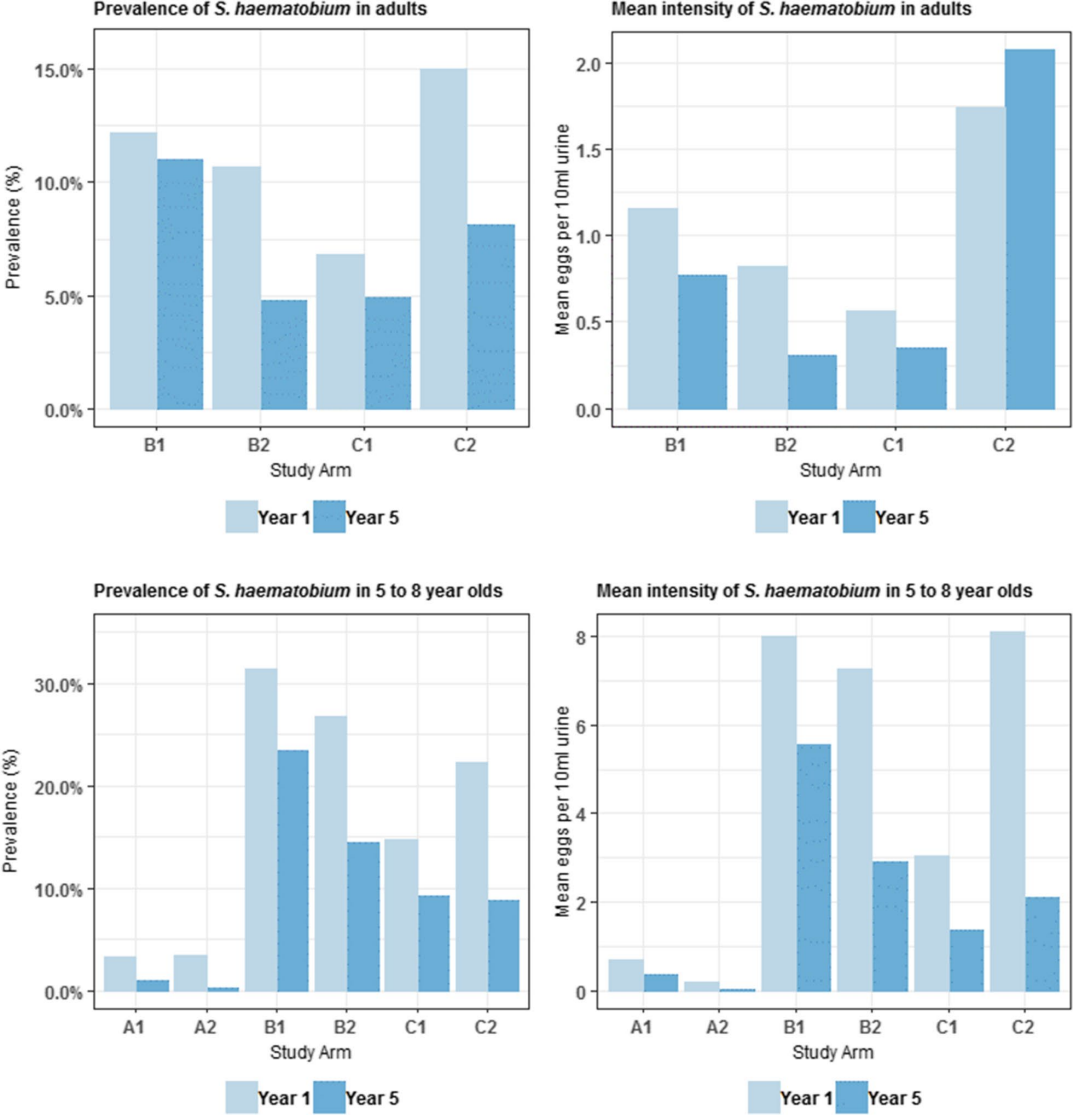
Prevalence of *Schistosoma haematobium* infection rates at baseline and Year 5 by study arm (adults and first year student’s cross-section only). The change in prevalence and intensity among adults (20–55 years-old) and first year students (5–8 years-old) across all study arms from baseline to Year 5 is shown

When separated by treatment group, prevalence, and intensity (with exception of Group C2 in adults) reduced in both adults and first year students across all arms from baseline to Year 5 (Fig. 6). Of interest is the reduction in adult prevalence that is statistically significant in both B1 (12.2% to 11%) and B2 (10.7% to 4.8%) arms where SBT was given only (*P* <0.001). This would suggest that SBT alone has an impact on disease transmission.

## Discussion

The SCORE project commenced in Niger with the goal of providing an evidence base for programmatic decisions on how best to control urogenital schistosomiasis, and ultimately eliminate it as a public health problem. The initial aim of this study was to examine the impact of SBT, CWT and treatment holidays for the control of *S. haematobium* in villages with both high (≥ 21%) and moderate (10–24%) prevalence. In Year 3 the study was redesigned, however, to compare annual versus biannual treatment delivered by SBT or CWT.

The primary research question presented here is which PC strategy provided the greatest reduction in prevalence and intensity of *S. haematobium* infection among 9–12 year-olds after four years of intervention in the Niger River Valley. Biannual SBT treatment was found to be significantly more effective for reducing active schistosome infection than annual SBT in high prevalence areas (Arm B1 *vs* B2). There was no significant effect of treatment frequency, however, in areas with a low starting prevalence (Arm A1 *vs* A2), or in higher prevalence areas receiving CWT (Arm C1 *vs* C2).

The communities in Arm A demonstrate a different the pattern of change in prevalence than Arms B and C, which both show an unexpected spike in infection at Year 3. The villages in Arm A are located away from the Niger River, therefore the communities would be in a low transmission zone. It is likely that geographical location of the sites has affected the rate of reinfection, which in turn will influence treatment outcomes.

Although the purpose of the eligibility survey was to ensure a high enough starting prevalence to make comparisons between treatment arms, the eligibility survey had higher infection rates than the baseline parasitological survey. Several villages had high prevalence using Hemastix®, when assessed by urine filtration the prevalence was lower than 10% (Table 5). Some studies have shown that in very low prevalence settings, microhaematuria can be an unstable proxy for urogenital schistosomiasis [32, 33]. On the other hand, the sensitivity of microscopic egg detection can vary according to intensity of infection, day-to-day variation in egg secretion, time the sample was collected, and number of examined samples [34–38]. Another potential reason for the difference in dipstick and microscopic examination could be the fact that the eligibility survey was carried out in 13–14 year-olds and the parasitology survey among 9–12 year-olds. Some studies have shown that 13–14 year-olds harbour higher infection levels due to them not attending school and therefore more likely to be exposed to infection [39]. Other studies have showed that many cases of urogenital schistosomiasis stay undetected when the examination method is limited to urine filtration [40, 41].

**Table 5:** Prevalence of *S. haematobium* in western Niger using Haemastix reagent dipsticks (2010–2011)

District	No. of schools sampled	No. of people sampled	No. of schools eligible	No. of people sampled in eligible schools	Method: Dipstick (Eligibility survey among 13–14 year-olds)		Method: Urine filtration (Baseline data collection among 9–12 year-olds)	
					Prevalence (%)	95% CI	Prevalence (%)	95% CI
CUN5	3	150	1	50	58.00	na	4.00	na
DS1	1	50	1	50	54.00	na	32.00	na
DS3	1	50	1	50	30.00	na	8.00	na
Filingue	93	4528	44	2141	20.10	16.71–23.50	5.72	2.93–8.52
Kollo	87	4235	52	2558	42.43	36.35–48.51	26.68	19.72–33.64
Loga	54	2641	26	1281	13.75	12.03–15.47	1.70	0.78–2.62
Say	76	3568	31	1501	37.19	31.79–42.60	13.07	7.90–18.24
Tera	45	2242	41	2042	39.92	33.87–45.96	18.79	12.51–25.07
Tillaberi	32	1632	28	1399	38.12	27.22–49.03	24.29	13.10–35.49
Total	392	19,096	225	11,072				

*na* not applicable

Both age and sex influenced infection where the highest prevalence and intensities of infection occurred in males and among older adolescents, with infection decreasing in adulthood [42, 43]. There was no significant difference, however, in response to treatment approach by age and sex.

To evaluate the potential longer-term impact of the programme, we also examined infection in first-year students, who had not received treatment previously as they had only recently started attending school. *Schistosoma haematobium* infection in this group of children decreased significantly over four rounds of treatment, implying that the level of environmental transmission in these areas could also have been reduced.

Currently the justification for targeting adults in a schistosomiasis control programme is based on the prevalence of infection in SAC. A study in Nigeria, however, has shown that monitoring and evaluation of SAC was not a successful indicator of the burden of infection in adults [44]. Prevalence among adults will likely be driven by many local behavioural and cultural factors, and is, therefore, context specific. This makes it difficult to make a SAC infection threshold for switching to CWT. For this reason, adults were also sampled in this study. The findings here demonstrated that biannual CWT was not significantly more effective at reducing prevalence and intensity of infection. This lack of statistical significance may be attributed to the difference in starting prevalence of the two CWT groups where Group C1 was 14.3% and C2 was 23.4%. Indeed, it has been modelled by Turner et al. [16] that the higher the pre-control burden in adults, the larger the benefit of switching to CWT. Interestingly, there was a significant reduction in prevalence of *S. haematobium* infection among adults even in Group B implying that the rate of transmission in the overall community had been decreased, even where only school children have been treated. These findings of reduction in infection among adults even in SBT communities were also seen in Mozambique [45].

Research in Niger has shown that the full economic delivery cost of SBT in 2005-2006 was $0.76 and CWT was $0.46. Including only the programme costs the figures were $0.47 and $0.41 respectively [46]. Differences at the sub-district level were more marked. This is partly explained by the fact that a CDD treats 5.8 people for every one treated in school [46]. Given that prevalence decreased among adults in SBT areas and twice-yearly CWT did not have a significantly greater impact on prevalence reduction with respect to annual CWT, these findings have significant logistical and cost-saving implications for a national control programme.

The success of PC programmes may be influenced by a wide range of factors such as initial endemicity and transmission intensities in the local environment, treatment frequency, coverage and compliance, among others [47]. Coverage data among different age groups is an important key determinant for achieving programme targets. Although average treatment coverage in the Niger SCORE programme has been shown to be well above the recommended WHO 75% threshold, in some areas it was above 100% and therefore implies an unreliable denominator. This is a common challenge for national control programmes as population census’ are often out of date or inaccurate. There may have been other external issues that might have affected the success of the biannual treatment. For example, there may have been a considerable gap between coverage and compliance [48]. Even when the proportion of eligible people who received tablets reach a significant fraction of the target population (coverage), those ingesting all the tablets at the same time (compliance) may be a better indicator of how well PC is being implemented. In this study, SBT was directly observed as the survey teams were present in the schools during the treatment. For CWT, the CDD supervised consumption of the treatment. In addition, frequent migration of people particularly among fishing communities or the nomadic Peule communities, some of whom move between neighbouring countries, remain significant hurdles to the successful implementation of the control programme, particularly in terms of ensuring repeated treatment. Finally, there was an increase in prevalence seen across all Groups in Year 3. This is likely the consequence of severe flooding across the country in 2012, which would have increased water contact as well as affecting the snail population, in addition to subsequent migration.

There were limitations to the study. One was the treatment coverage, discussed above, particularly where adults presented for treatment at the school in the SBT arms. The other limitation was the differential in time periods between MDAs and parasitological surveys, which were not conducted precisely at once and six-monthly time-points due to the logistical challenge of conducting such large surveys and treatment in such a short time frame. It is not clear whether this contributed to the findings as it was difficult to account for this in the analysis.

The findings here show that with good coverage biannual SBT may be the best strategy for schistosomiasis control in high prevalence areas. In low transmission settings, however, the lack of statistical impact of two rounds of treatment indicate that it may not be possible to break transmission through PC alone and using other strategies such as health education, water, sanitation and hygiene (WaSH), and snail control, should also be considered [49, 50].

## Conclusions

The rationale behind biannual treatment was to reduce further the prevalence and intensity of infection, meaning a greater impact on schistosomiasis control. We conclude that twice yearly SBT treatment was effective in reducing *S. haematobium* in high prevalence areas, with reported high treatment coverage. Biannual SBT in low prevalence settings and CWT in high prevalence areas did not have a significant impact on infection levels; therefore, it might not be cost-effective to treat biannually in such areas but focus on more targeted treatment in persistent hot spot areas, where infection remains high despite years of treatment. Finally, there was a significant reduction in prevalence observed in non-targeted age groups in the SBT communities. This suggests that SBT has an impact at reducing transmission potential in the community. The effect this has on adults and other non-targeted age groups should be investigated further as there would be significant cost implications in maintaining SBT strategies only.

## Data Availability

Data supporting the conclusions of this article are included within the article. The datasets used and analysed during this study are available from the corresponding author upon reasonable request.
